# Data on artificial neural network and response surface methodology analysis of biodiesel production

**DOI:** 10.1016/j.dib.2020.105726

**Published:** 2020-05-20

**Authors:** A.A. Ayoola, F.K. Hymore, C.A. Omonhinmin, P.O. Babalola, E.O. Bolujo, G.A. Adeyemi, R. Babalola, O.A. Olafadehan

**Affiliations:** a Chemical Engineering Department, Covenant University, Ota, Ogun State, Nigeria; b Regent University College of Science and Technology, Accra, Ghana; c Biological Sciences Department, Covenant University, Ota, Ogun State, Nigeria; d Mechanical Engineering Department, Covenant University, Ota, Ogun State, Nigeria; e Petroleum Engineering Department, Covenant University, Ota, Ogun State, Nigeria; f Chemical/Petrochemical Engineering Department, Akwa Ibom State University, Nigeria; g Chemical and Petroleum Engineering Department, University of Lagos, Nigeria

**Keywords:** ANN, Biodiesel, KOH, NaOH, RSM, Waste soybean oil

## Abstract

The biodiesel production from waste soybean oil (using NaOH and KOH catalysts independently) was investigated in this study. The use of optimization tools (artificial neural network, ANN, and response surface methodology, RSM) for the modelling of the relationship between biodiesel yield and process parameters was carried out. The variables employed in the experimental design of biodiesel yields were methanol-oil mole ratio (6 – 12), catalyst concentration (0.7 – 1.7 wt/wt%), reaction temperature (48 – 62°C) and reaction time (50 – 90 min). Also, the usefulness of both the RSM and ANN tools in the accurate prediction of the regression models were revealed, with values of R-sq being 0.93 and 0.98 for RSM and ANN respectively.

Specifications table

**Table:** 

Subject area	Energy, Engineering, Environmental Science
More specific subject area	Renewable Energy, Sustainability and the Environment, Engineering, Environmental Engineering
Type of data	Table, graph, figure
How data were acquired	Experimental design on transesterification reaction, the use of ANN algorithm, use of Minitab 17 software, model
Data format	Raw, analysed
Parameters for data collection	The variables employed in the generation of biodiesel yield data were methanol-oil mole ratio (6 – 12), catalyst concentration (0.7 – 1.7 wt/wt%), reaction temperature (48 – 62°C) and reaction time (50 – 90 minutes). Box-Benkehn BB(4) design in Minitab 17 environment was used for the design of experiments, ANN algorithm and RSM software were used for optimization studies. The esterification process of free fatty acid (FFA) removal involved the addition of 40 mL of a mixture of 25 mL isopropyl alcohol and 15 mL benzene solution to waste soybean oil (heated to 55°C), as well as the addition of 2 drops of phenolphthalein and the mixture was then titrated with 0.1 M KOH. Removal of water was done by heating the esterified oil at 110°C for 20 min.
Description of data collection	RSM analysis was done through the adoption of three (3) continuous factors: one (1) number of categorical factor, one (1) number of block and one (1) number of replicate for the plots of biodiesel yields and prediction of statistical models, analysis of *Sum of Errors coefficients* (SE coefficients), *p values* and *F values*. ANN algorithms were sectioned into data division (random), training (Levenberg Marquardt) and performance (mean squared errors). The 27 samples from the Box-Benkehn BB(4) design were distributed into training, validation and testing in the proportion of 19 samples (70%), 4 samples (15%) and 4 samples (15%) respectively. Plots of regression (training, validation, test and overall), mean squared error and error histogram were made using ANN algorithm.
Data source location	Department of Chemical Engineering, Covenant University, Ota, Nigeria.
Data accessibility	With the article

## Value of the data

• The data aided the determination of the quality level and efficiency of the two processes investigated in biodiesel production.
• The data are useful to authors in the field of renewable energy (at the global level). This is because the data revealed the processing conditions for the production of high yield and high-quality WSO-biodiesel.
• The ANN and RSM data could be used to predict reliable models that comparatively connect yields, processing conditions and the quality of yields (in terms of statistical tools).
• The ANN and RSM data could be used to improve the biodiesel production process by focusing on the operating conditions that gave high-quality value of biodiesel.
• The data showed the comparative assessment of KOH and NaOH catalysed processes (under the same operating conditions) during the WSO biodiesel process. This is a piece of important information in the consideration of a more suitable catalyst for WSO biodiesel process.

## 1 Data Description

**Fig. 1:**
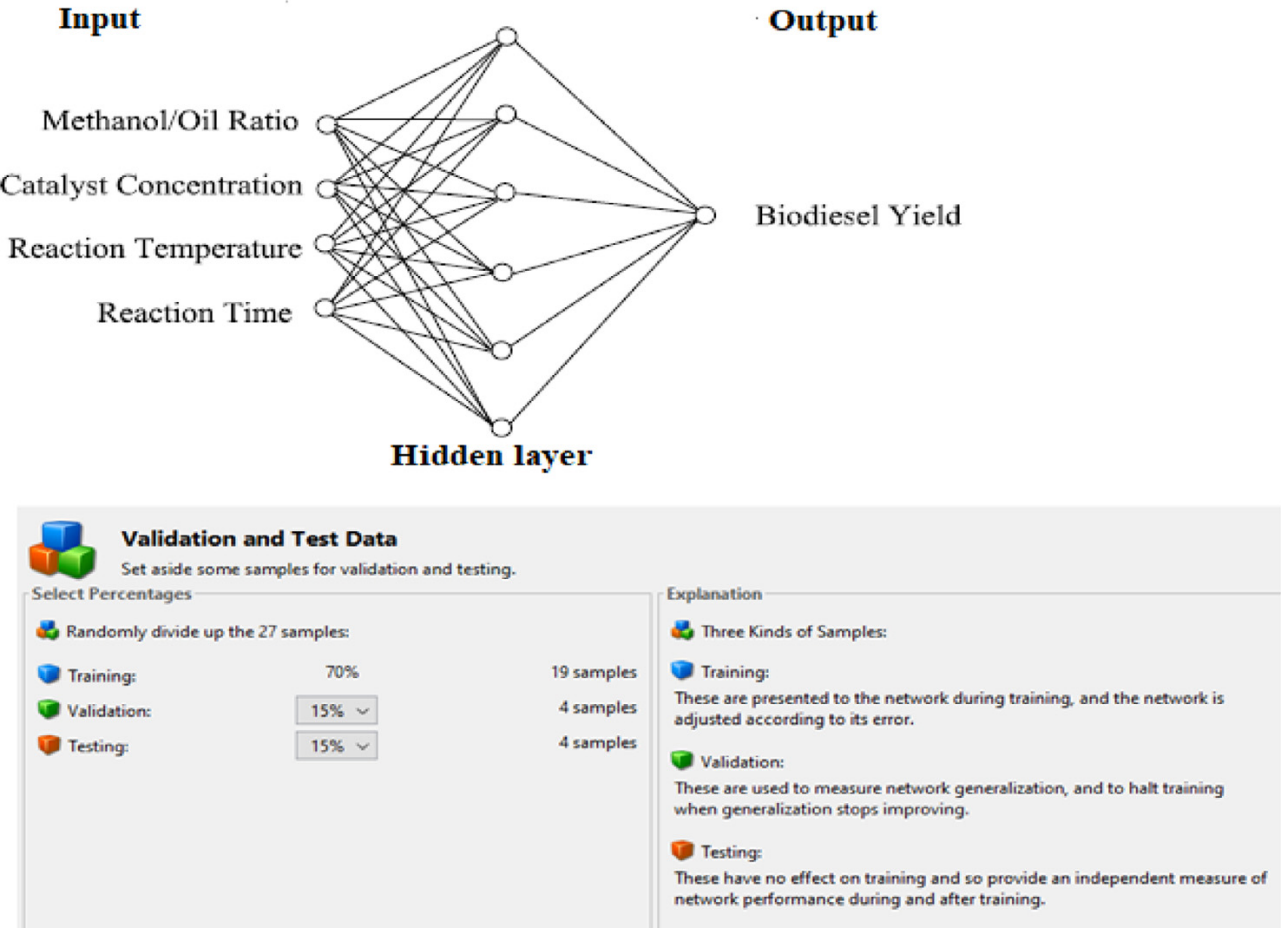
ANN Architecture

Fig. 1 depicts the ANN Architecture in which the 27 samples (from the experimental design) were distributed into training, validation and testing in the proportion of 19 samples (70%), 4 samples (15%) and 4 samples (15%) respectively. The experimental design (using Box-Behnken BB(4)) for WSO biodiesel production at three levels and four process parameters (X_1_, X_2_, X_3_and X_4_) is presented in Table 1. However, when ANN technique was applied, these experimental input parameters were re-assigned *α*_m_, *α*_c_, *α*_T_ and *α*_t_ to represent the mole ratio of methanol to oil, concentration of catalyst, temperature of reaction and time of reaction respectively. For ease of application of ANN algorithm, these four input parameters and the yields were computed by dividing each column of the actual process variables by the highest value in the column so as to have a range of zero (0) to one (1) values.

**Table 1:** Experimental design for WSO biodiesel production using Box-Behnken BB(4)

Process parameter	Notation	Levels		
		-1	0	+1
Methanol to oil mole ratio	X_1_	6:1	9:1	12:1
Concentration of catalyst, wt/wt% oil	X_2_	0.7	1.2	1.7
Temperature of reaction, °C	X_3_	48	55	62
Time of reaction, minutes	X_4_	50	70	90

Table 2 shows the process parameters and the WSO biodiesel yield obtained from RSM and ANN tools.

**Table 2:** Process variables and biodiesel yield (response) obtained from RSM and ANN techniques

S/N	Process variable				Yield			
	*α*_m_	*α*_c_	*α*_T_	*α*_t_	RSM (NaOH)	ANN (NaOH)	RSM KOH	ANN (KOH)
1	1.00	1.00	0.89	0.78	0.91	0.93	0.82	0.84
2	1.00	0.41	0.89	0.78	0.93	0.92	0.94	0.91
3	0.50	1.00	0.89	0.78	0.88	0.89	0.89	0.87
4	0.50	0.41	0.89	0.78	0.89	0.89	0.98	0.98
5	0.75	0.71	1.00	1.00	0.98	0.97	0.85	0.84
6	0.75	0.71	1.00	0.56	0.92	0.94	0.84	0.87
7	0.75	0.71	0.77	1.00	0.92	0.92	0.88	0.89
8	0.75	0.71	0.77	0.56	0.94	0.92	0.84	0.87
9	0.75	0.71	0.89	0.78	0.94	0.94	0.91	0.87
10	1.00	0.71	0.89	1.00	0.93	0.93	0.84	0.85
11	1.00	0.71	0.89	0.56	0.91	0.92	0.92	0.91
12	0.50	0.71	0.89	1.00	0.89	0.89	0.88	0.88
13	0.50	0.71	0.89	0.56	0.88	0.88	0.89	0.90
14	0.75	1.00	1.00	0.78	0.95	0.97	0.78	0.82
15	0.75	1.00	0.77	0.78	0.90	0.93	0.82	0.82
16	0.75	0.41	1.00	0.78	0.93	0.94	0.83	0.86
17	0.75	0.41	0.77	0.78	0.91	0.91	0.98	0.99
18	0.75	0.71	0.89	0.78	0.94	0.94	0.90	0.87
19	1.00	0.71	1.00	0.78	0.93	0.90	0.85	0.83
20	1.00	0.71	0.77	0.78	0.91	0.91	0.93	0.91
21	0.50	0.71	1.00	0.78	0.91	0.91	0.86	0.88
22	0.50	0.71	0.77	0.78	0.88	0.88	0.93	0.91
23	0.75	1.00	0.89	1.00	0.93	0.94	0.88	0.85
24	0.75	1.00	0.89	0.56	0.92	0.93	0.80	0.82
25	0.75	0.41	0.89	1.00	0.93	0.93	0.95	0.92
26	0.75	0.41	0.89	0.56	0.92	0.93	0.93	0.91
27	0.75	0.71	0.89	0.78	0.94	0.94	0.91	0.87

**Fig. 2:**
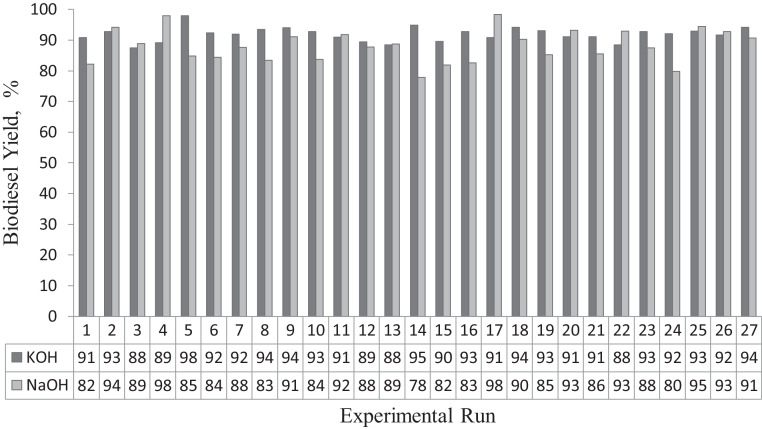
WSO biodiesel yields from KOH and NaOH catalysed processes

Fig. 2 shows the comparative analyses of the WSO biodiesel yields from KOH and NaOH catalysed processes. Eqs. 1, 2 show the respective RSM models that relate the biodiesel yields and the four process variables investigated using KOH and NaOH catalysts. The fitness and reliability of the models were revealed by the high values of ***R-sq*** and ***R-sq (adj.)***, low values of probability value (***p ≤ 0.05*** at significant level), the low values of ***Sum of Errors (SE) coefficient*** and high values of ***F***, as presented in Table 3, Table 4 for the statistical analyses of the RSM models for WSO biodiesel production using KOH and NaOH catalysts respectively.

**Table 3:** Statistical analysis of the RSM model for WSO biodiesel production using KOH catalyst

Term	Coefficient	SE Coefficient	F	p
Constant	73.9378	6.11326	35.169	0.000000
Linear				
X_1_	6.5672	0.55554	139.744	0.000000
X_4_	-0.5117	0.15424	11.006	0.003831
Square				
X_1_X_1_	-0.3379	0.03065	121.552	0.000000
X_2_X_2_	-5.9667	1.00819	35.025	0.000013
X_4_X_4_	-0.0010	0.00069	2.132	0.161516
Interaction				
X_2_X_3_	0.2521	0.04434	32.314	0.000022
X_3_X_4_	0.0125	0.00232	29.144	0.000040

**Table 4:** Statistical analysis of the RSM model for WSO biodiesel production using NaOH catalyst

Term	Coefficient	SE Coefficient	F	p
Constant	-48.6299	3.0973	27.632	0.003400
Linear				
X_2_	-56.6067	0.7498	117.043	0.089000
X_3_	5.6742	0.2134	10.806	0.063291
X_4_	1.0185			
Square				
X_1_X_1_	0.1080	0.06885	91.791	0.03050
X_3_X_3_	-0.0647	2.0097	32.505	0.00496
X_4_X_4_	-0.0050	0.00092	1.094	0.76270
Interaction				
X_1_X_4_	0.0321	0.05304	30.147	0.00022
X_2_X_3_	0.8400	0.03772	26.375	0.00921

**Fig. 3:**
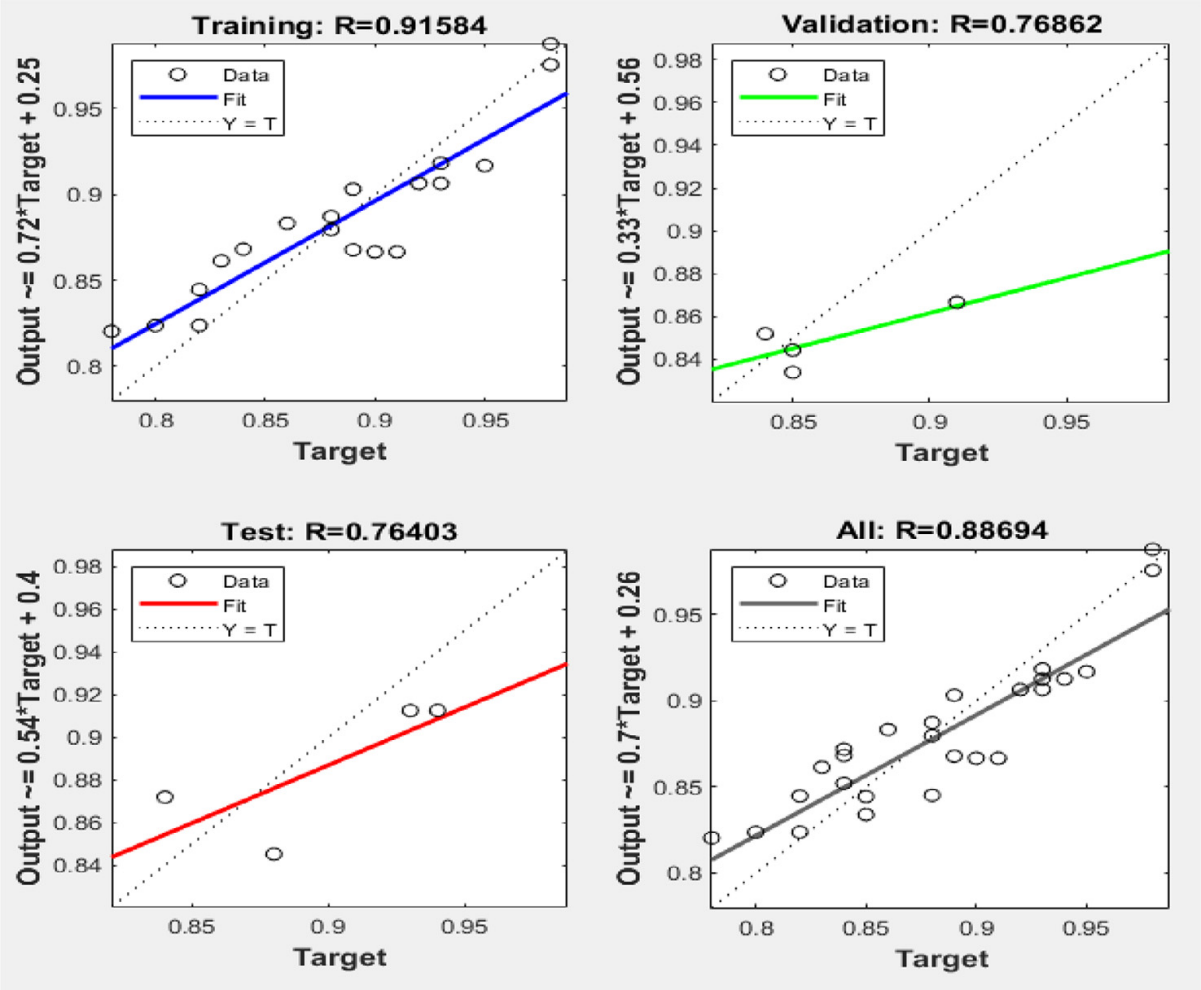
Plots of regression: (i) training, (ii) validation (iii) test (iv) overall for WSO biodiesel production via KOH catalysed process

**Fig. 4:**
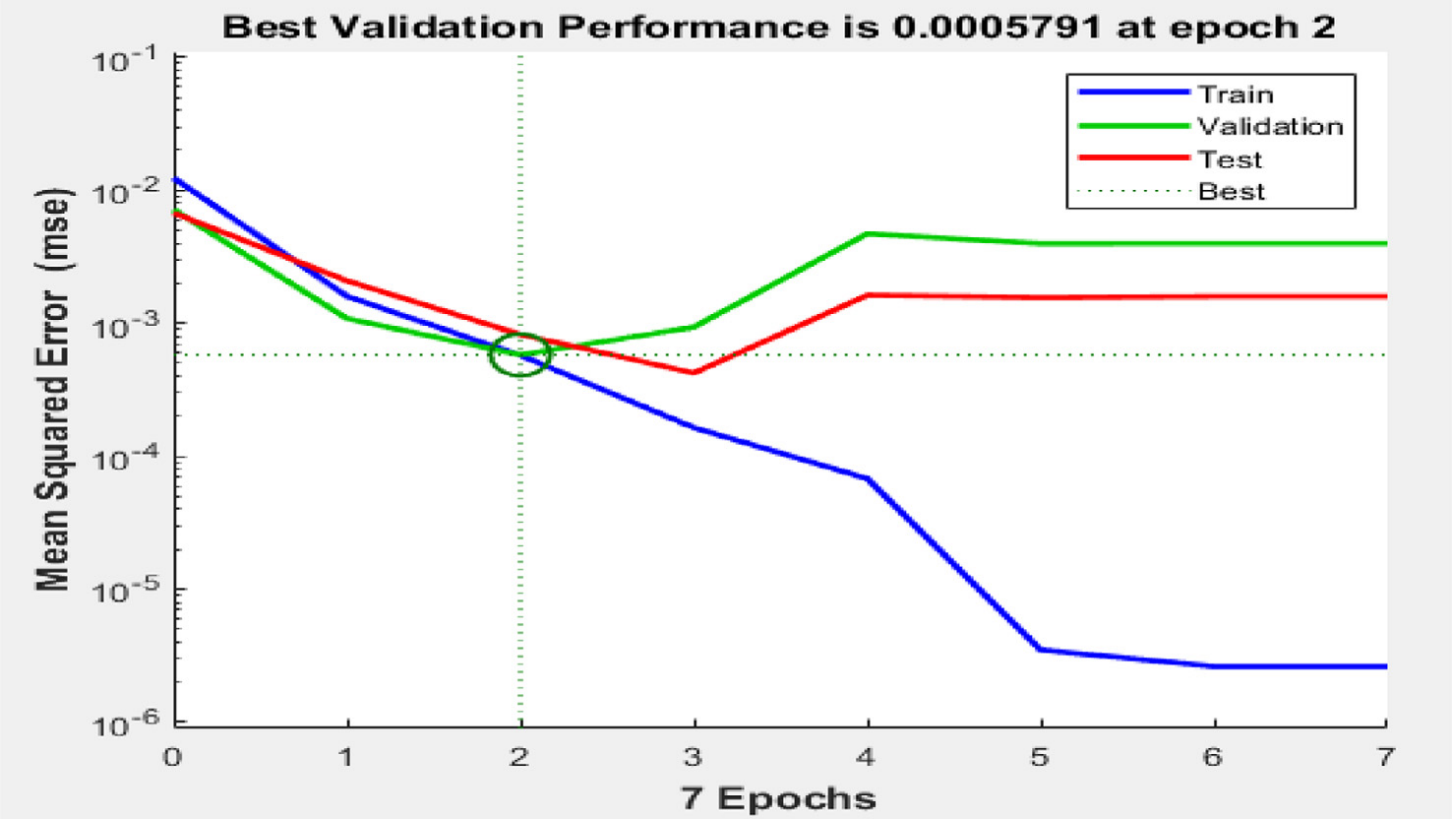
Mean squared error for biodiesel production from WSO using KOH catalyst

**Fig. 5:**
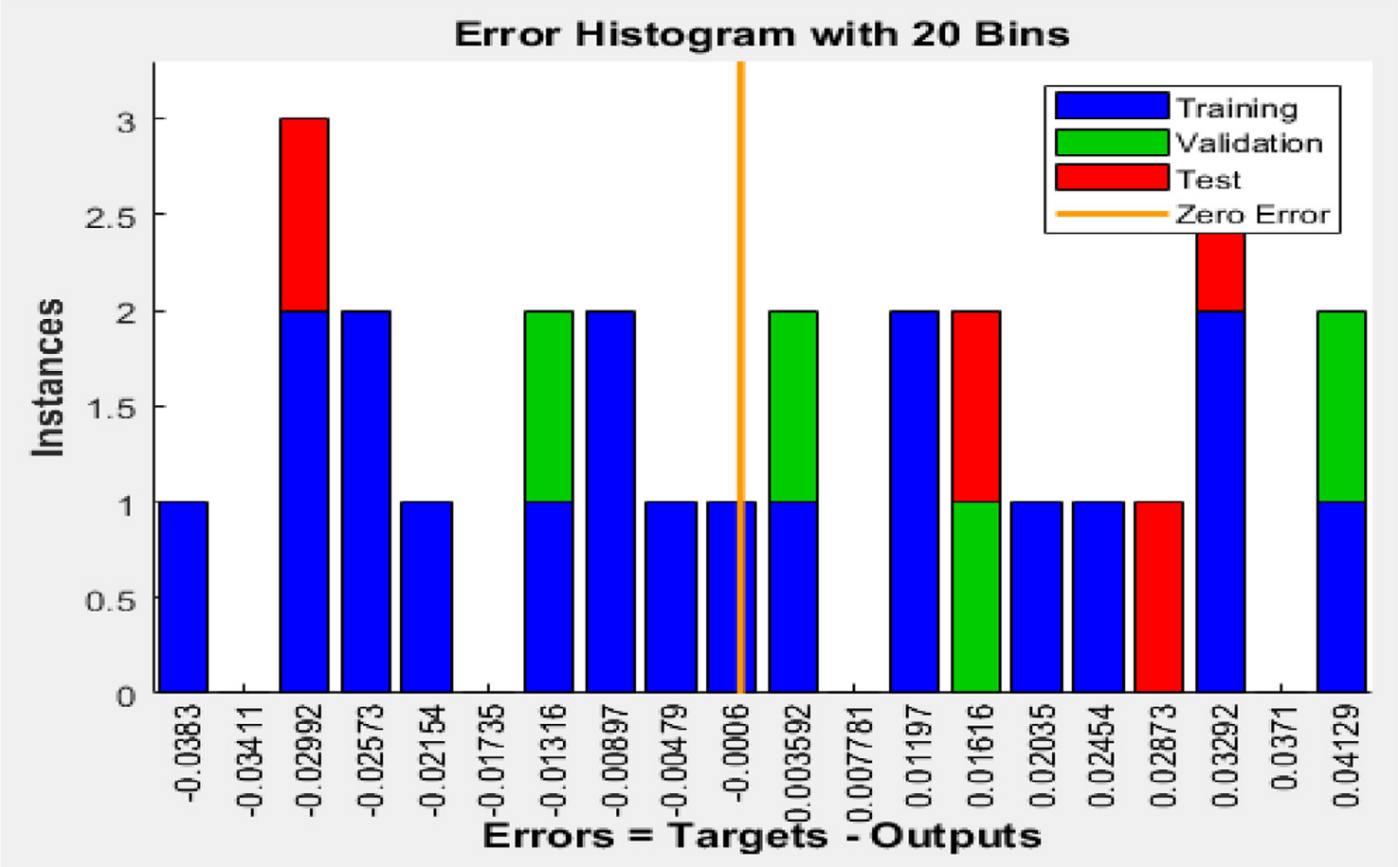
Error histogram for biodiesel production from WSO using KOH catalyst

**Fig. 6:**
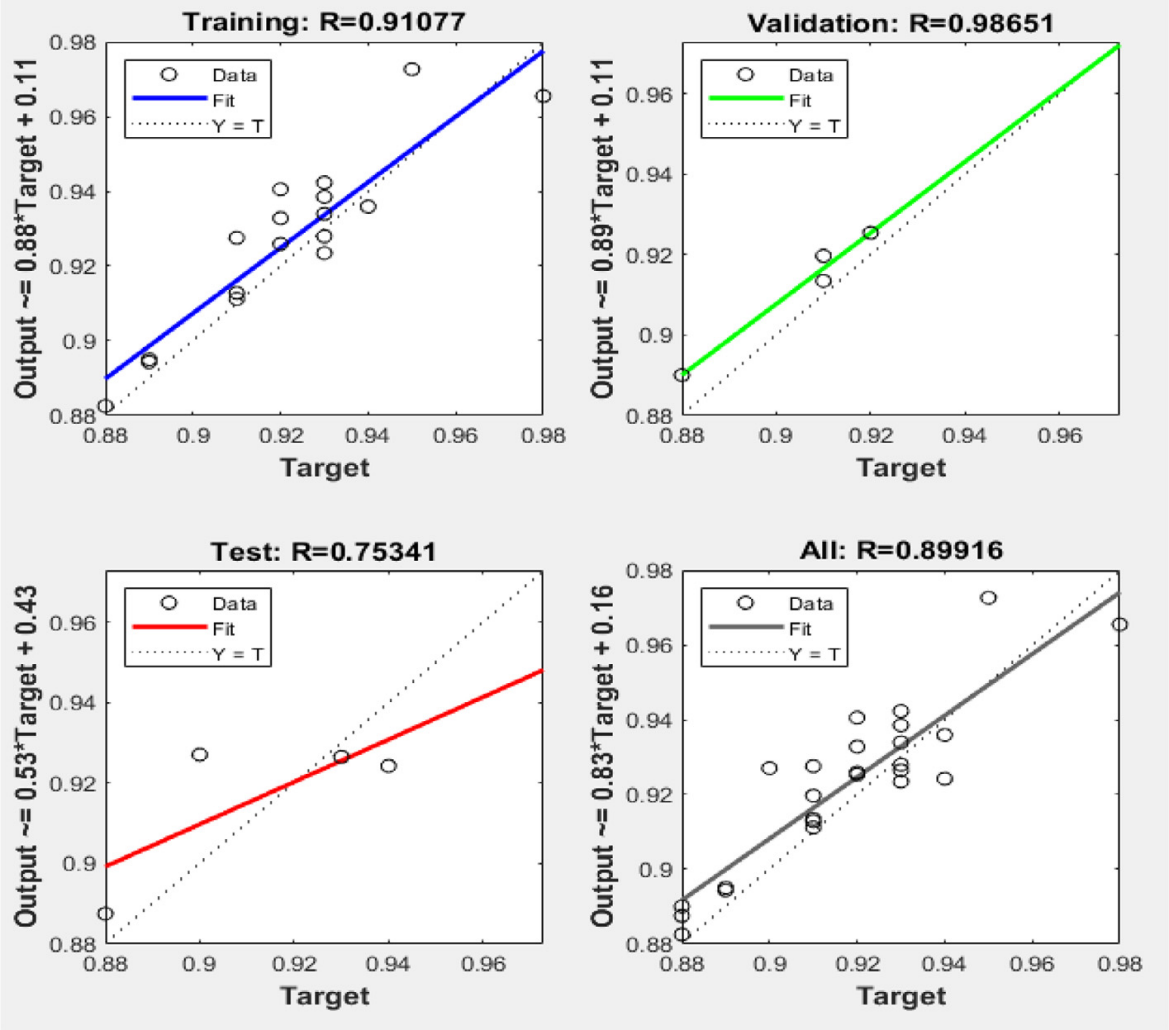
Plots of regression: (i) training, (ii) validation (iii) test (iv) overall for NaOH catalysed WSO- biodiesel production

**Fig. 7:**
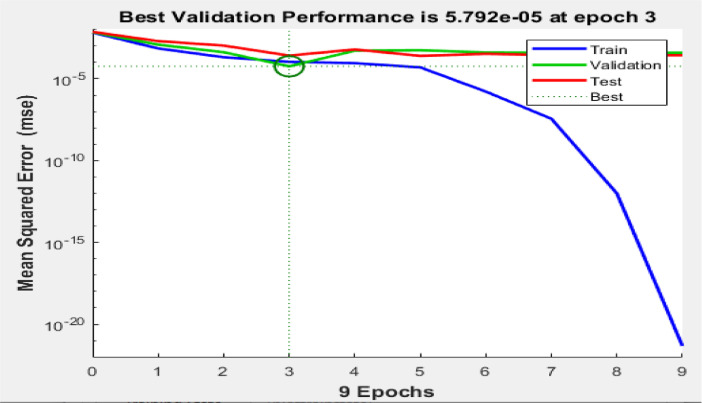
Mean squared error for biodiesel production from WSO using NaOH catalyst

**Fig. 8:**
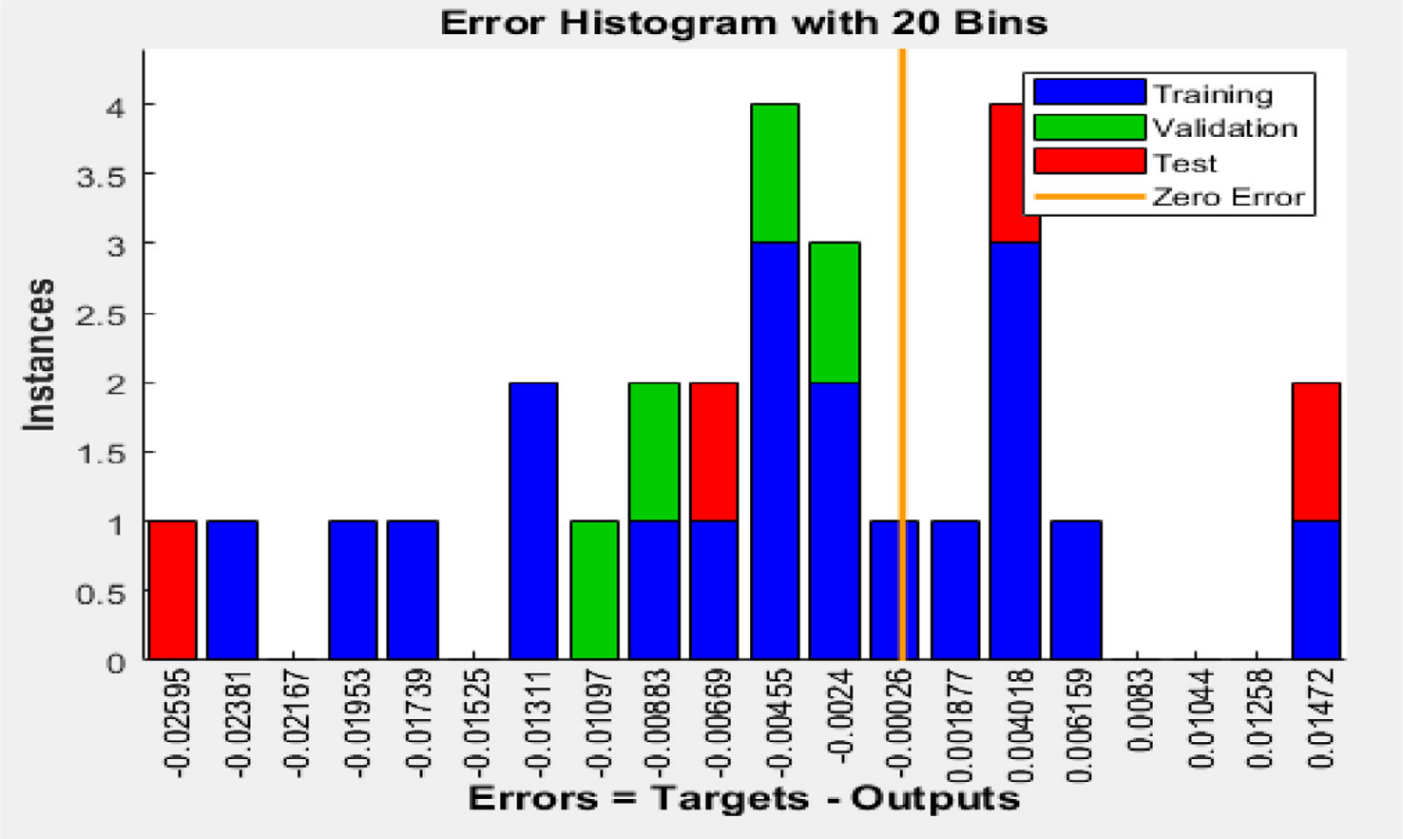
Error histogram for biodiesel production from WSO using NaOH catalyst

Fig. 3, Fig. 4, Fig. 5 reveal the respective plots of regression, mean squared error and error histogram for WSO biodiesel production using KOH catalyst. Fig. 6, Fig. 7, Fig. 8 reveal the respective plots of regression, mean squared error and error histogram for WSO biodiesel production using NaOH catalyst. The best ANN topology could be observed at the least mean squared error value of approximately zero (0) and a correlation coefficient of one (1). The training of the network was terminated at the overshooting point.

(1) $$\begin{array}{ccc} & & {\left(\mathrm{WSO}\mathrm{biodiesel}\mathrm{yield}\right)}_{\mathrm{KOH}}\\ & & \;=73.9378+6.5672\;X_{1}-0.5116\;X_{4}-0.3379\;X_{1}X_{1}-5.9666\;X_{2}X_{2}\\ & & \;+\;0.2520\;X_{2}X_{3}-0.0089\;X_{3}X_{3}+\;0.0125\;X_{3}X_{4}-0.0010\;X_{4}X_{4}\\ & & \;R-\text{sq}=93.99\%\;\;\;\;\;R-\text{sq}\left(\text{adj}\right)=91.31\% \end{array}$$

(2) $$\begin{array}{ccc} & & {\left(\mathrm{WSO}\mathrm{biodiesel}\mathrm{yield}\right)}_{\mathrm{NaOH}}\\ & & \;=-48.6299-56.6067\;X_{2}+5.6742\;X_{3}+1.0185\;X_{4}+0.1080\;X_{1}X_{1}-0.0321\;X_{1}X_{4}\\ & & \;+0.84\;X_{2}X_{3}-0.0647\;X_{3}X_{3}-0.0050\;X_{4}X_{4}\\ & & \;R-\text{sq}=80.18\%\;\;\;\;\;R-\text{sq}\left(\text{adj}\right)=71.37\% \end{array}$$

## 2 Experimental Design, Materials, and Methods

The pre-treatment of WSO involves the removal of impurities, free fatty acid (FFA) and water to prevent low yield of biodiesel (and even formation of other products) during transesterification reaction [1], [2], [3], [4], [5], [6]. Hence, WSO was pre-treated before the transesterification process was carried out. Impurities were removed through filtration process (using the industrial sieve of 50 *μ*m pore size), FFA was removed through the esterification process which involved mixing 25 mL isopropyl alcohol with 10 mL benzene. 40 mL of this solution was then added to WSO and heated to 55°C. 2 drops of phenolphthalein were added to the mixture and then titrated with 0.1 M KOH until a permanent pale pink colouration was observed. Removal of water was done by heating the esterified oil at 110°C for 20 min [7,8].

Box-Benkehn BB(4) experimental design (through the application of Minitab 17 software) was used for the design of experiments. The software was also used for statistical analysis, which involved the plots of experimental biodiesel yields and statistical modelling. The procedures used for the transesterification of the waste soybean oil using methanol and the two forms of catalysts (KOH and NaOH) separately were exhaustively outlined in Ayoola et al. [9,10].

The 27 samples from the experimental design were distributed into training, validation and testing in the proportion of 19 samples (70%), 4 samples (15%) and 4 samples (15%) respectively. To generate the R-sq values and least error conditions, the plots of regression (training, validation, test and overall), mean squared error and error histogram were made using the ANN algorithm.

## Declaration of Competing Interest

The authors declare that they have no known competing financial interests or personal relationships which have, or could be perceived to have, influenced the work reported in this article.

## Appendix Supplementary materials

Image, application 1
